# Enhancement of anti-leukemia activity of NK cells *in vitro* and *in vivo* by inhibition of leukemia cell-induced NK cell damage

**DOI:** 10.18632/oncotarget.6529

**Published:** 2015-12-09

**Authors:** Roberto Arriga, Sara Caratelli, Andrea Coppola, Giulio Cesare Spagnoli, Adriano Venditti, Sergio Amadori, Giulia Lanzilli, Davide Lauro, Patrizia Palomba, Tommaso Sconocchia, Maria Ilaria Del Principe, Luca Maurillo, Francesco Buccisano, Barbara Capuani, Soldano Ferrone, Giuseppe Sconocchia

**Affiliations:** ^1^ Department of Systems Medicine, University of Rome “Tor Vergata”, Rome, Italy; ^2^ Laboratory of Tumor Immunology and Immunotherapy, Institute of Translational Pharmacology, Department of Biomedicine, National Research Council (CNR), Rome, Italy; ^3^ Institute for Surgical Research and Hospital Management, Department of Biomedicine, University of Basel, Basel, Switzerland; ^4^ Hematology, Department of Biomedicine and Prevention, University of Rome “Tor Vergata”, Rome, Italy; ^5^ Departments of Surgery and Orthopedic Surgery, Massachusetts General Hospital, Harvard Medical School, Boston, MA, USA

**Keywords:** NK cell, acute myeloid leukemia, CD16, TIMP3, NK cell abnormalities

## Abstract

Acute myeloid leukemia (AML) cells induce, *in vitro*, NK cell abnormalities (NKCAs) including apoptosis and activating receptor down-regulation. The potential negative impact of AML cells on the therapeutic efficacy of NK cell-based strategies prompted us to analyze the mechanisms underlying NKCAs and to develop approaches to protect NK cells from NKCAs. NKCA induction by the AML leukemia cells target a subpopulation of peripheral blood NK cells and is interleukin-2 independent but is abrogated by a long-term culture of NK (LTNK) cells at 37°C. LTNK cells displayed a significantly enhanced ability to damage AML cells *in vitro* and inhibited the subcutaneous growth of ML-2 cells grafted into CB17 SCID mice. Actinomycin D restored the susceptibility of LTNK cells to NKCAs while TAPI-0, a functional analog of the tissue inhibitor of metalloproteinase (TIMP) 3, inhibits ML-2 cell-induced NKCAs suggesting that the generation of NK cell resistance to NKCAs involves RNA transcription and metalloproteinase (MPP) inactivation. This conclusion is supported by the reduced susceptibility to AML cell-induced NKCAs of LTNK cells in which TIMP3 gene and protein are over-expressed. This information may contribute to the rational design of targeted strategies to enhance the efficacy of NK cell-based-immunotherapy of AML with haploidentical NK cells.

## INTRODUCTION

Growing clinical evidence indicates that immunotherapy with NK cells is useful in the treatment of myeloid leukemia. However, in the majority of patients, the efficacy of this therapy is limited since the clinical responses are not always complete, or the disease recurs.[1–7] These clinical findings have prompted investigators to characterize the interactions of NK cells with AML cells, since the resulting information may contribute to the rationale design of strategies to optimize the efficacy of NK cell-based immunotherapy of AML. Convincing experimental and clinical evidence indicates that AML cells damage NK cells; this damage reduces the ability of NK cells to control AML cell growth and provides AML cells with the capacity to escape from NK cell immunity. The damages induced by AML cells to NK cells both *in vitro* and *in vivo* are referred to as NKCAs; they include apoptosis[8], defective expression and function of natural cytotoxic receptors (NCRs), [9, 10] and CD16 down-regulation. Scant information is available about the mechanism(s) underlying the induction of NKCAs by AML cells; although, this information may contribute to the rationale design of strategies to inhibit or counteract their induction. Therefore, in this study guided by the notion that MMP chemical inhibitors reverse CD16 down-regulation,[11] induced by AML cells, we have investigated whether MMP endogenous inhibitors are involved in the inhibition of AML cell-induced CD16 down-regulation. Furthermore, because of the association of CD16 cross-linking by mAb with the induction of NK cell apoptosis,[12] we have investigated the role of CD16 in the induction of AML-cell induced NK cell apoptosis and depletion. Lastly, taking advantage of the information generated by these experiments, we have developed a strategy to counteract the induction of NKCAs by leukemia cells.

## RESULTS

### NKCA induction by AML cells

**Figure 1:**
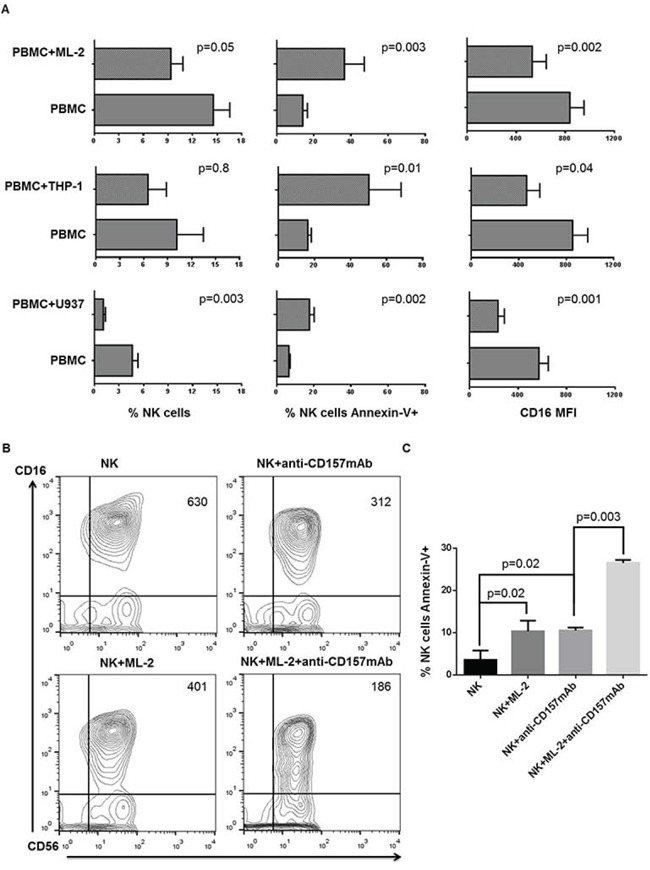
Human AML cell-induced NKCAs involves CD16 antigen Panel **A.** PBMCs from healthy donors were cultured for 3 days in the presence of IL-2. Cells were then harvested and incubated with AML cells at a ratio of 4 PBMCs to 1 AML cell. PBMCs incubated under the same experimental conditions, but without AML cells were used as controls. Following a 5-hour incubation at 37°C, cells were stained with FITC-annexin-V, PE-anti-CD16, PE-Cy5-anti-CD3, APC-anti-CD56 and analyzed utilizing a 2-laser flow cytometer. We assessed the effects of AML cells on CD16 mean fluorescence intensity (MFI), NK cell apoptosis, and NK cell depletion by designing an electronic gate on CD16+CD56+CD3- cells. This figure shows data obtained from 6 experiments independently performed. Panel **B.** Following a 3-day activation with IL-2, PBMCs were harvested, and NK cells were negatively sorted using immunomagnetic beads. NK cells were then incubated in the absence (panel B, upper left) or presence (panel B, lower left) of ML-2 cells. NK cells were incubated at room temperature for 30 min in the presence of the indicated anti-CD157 mAb with or without ML-2 cells then washed. Quadrant numbers indicate CD16 MFI. Following a 5-hour incubation CD16 and CD56 antigens and annexin-V were evaluated by flow cytometry. Panel **C.** shows differential expression of annexin-V on NK cells stimulated as indicated. This figure shows a representative experiment out of 3 performed with similar results.

Incubation of peripheral blood mononuclear cells (PBMCs) with the human AML cell line, ML-2, for 5 hours at 37°C induced: 1) CD16 down-regulation on NK cells; 2) apoptosis of NK cells as indicated by an increased frequency of Annexin-V+ NK cells as compared to the PBMCs incubated without the leukemic cell line and 3) depletion of NK cells as shown by a reduction in their number as compared to that in PBMCs incubated without the leukemia cell line. Similar results were obtained when the AML cell lines THP-1 and U937 were used; although, with some differences in the extent of changes. THP-1 cells were significantly less potent inducers of NKCAs than ML-2 and U937 cell lines (Figure 1A). The latter two cell lines did not differ from each other. The extent of the NKCAs induced by leukemia cells was markedly increased when NK cells incubated with leukemia cells were activated by cross-linking of CD16 mediated by its interaction with the Fc fragment of the CD157-specific mAb SY11B5. CD157 is expressed on leukemia cells but is not detectable on NK cells. These findings raise the possibility that CD16 plays a role in the induction of NKCAs by leukemic cells.

### CD16 involvement in the induction of NKCAs by AML cells

To investigate a cause-effect relationship between CD16 down-regulation and induction of NKCAs by leukemia cells, CD16 was cross-linked by incubating IL-2 stimulated short term NK (STNK) cells for 5 hours at 37°C with ML-2 cells coated with the CD157-specific mAb SY11B5. Although with some differences in the degree of changes, mAb SY11B5 enhanced ML-2 cell-induced CD16 down-regulation (Figure 1 panel B) and extent of apoptosis (Figure 1 panel C). The highest level of NKCAs was observed when NK cells were incubated with ML-2 cells and mAb SY11B5. Similar results were also obtained with U937 cells (data not shown). These findings are compatible with the possibility that CD16 antigen plays a role in the induction of NKCAs by leukemia cells. Additional evidence in support of this possibility is provided by CD16 higher expression on STNK than on LTNK cells (data not shown).

### Generation by long-term in vitro culture of NK cell resistance to leukemia cell-induced NKCAs

**Figure 2:**
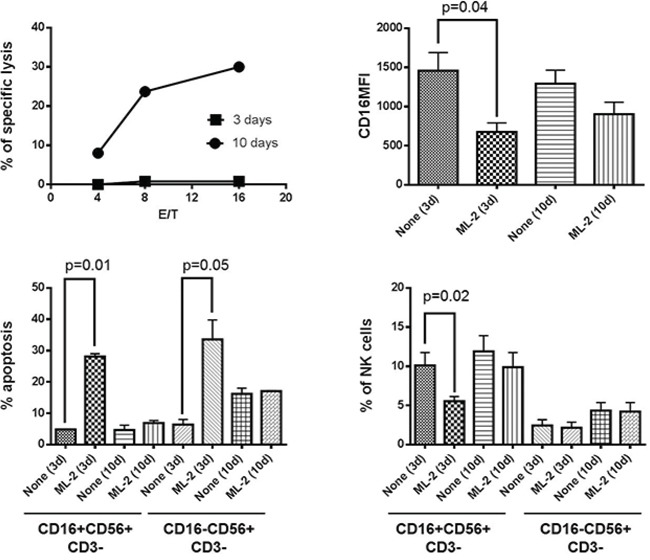
Long-term culture condition protects NK cells, in vitro, from ML-2 cell-induced NKCAs Following a 3 or a 10-day activation with IL-2, NK cells were incubated for 4 hours at 37°C with ^51^Cr-labelled ML-2 cells at the indicated E/T cell ratio (upper, left panel). For NKCAs: IL-2 stimulated PBMCs were cultured in the absence or presence of ML-2 at 4:1 E/T ratio. CD16MFI of NK cells (upper, right panel), percentage of apoptosis (lower, left panel) and percentage of NK cells (lower, right panel) were evaluated in the CD16+NK and CD16-NK cell subsets by flow cytometry.

To determine whether the duration of IL-2 incubation with NK cells affected their susceptibility to NKCAs, we investigated the effect of ML-2 cells on STNK and LTNK cell cultures. Figure 2 shows that STNK cells were susceptible to AML cell-induced NKCAs as indicated by a marked CD16 down-regulation (Figure 2, upper, right panel), depletion of CD16^+^ cells (Figure 2, lower, right panel) and induction of apoptosis of CD16+ and CD16- NK cells (Figure 2, lower, left panel). In contrast, LTNK cells that displayed a strong lytic activity (Figure 2, upper left, panel) were resistant to leukemia cell-induced NKCAs. This conclusion is indicated by the lack of significant difference between NK cells incubated with ML-2 and control NK cells incubated without ML-2 cells to the extent of CD16 down-regulation as well as cell apoptosis. These results indicate that long-term *in vitro* culture of NK cells reduces their susceptibility to AML cell-induced NKCAs. Similar results were obtained also when STNK and LTNK cells were tested for their ability to lyse the leukemia cell lines THP-1 and U937 and their susceptibility to induction of NKCAs by these leukemia cell lines (data not shown).

**Figure 3:**
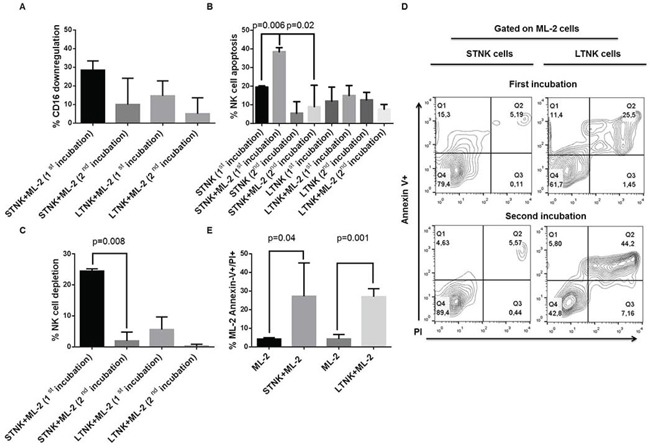
ML-2 cells reduces, *in vitro*, the frequency of short-term NK effector cells with anti-leukemia activity IL-2 post-activated STNK and LTNK cells were subjected to a 2 consecutive rounds of incubation with ML-2 cells at a ratio of 4 STNK or LTNK cells to 1 ML-2 cell at 37°C. Following a 5-hour and an 18-hour incubation of STNK and LTNK cells with ML-2 cells, cell aliquots were harvested and stained with FITC-annexin-V, PI, PE-anti-CD16, and APC-anti-CD56 and analyzed by flow cytometry for CD16 down-regulation panel **A.**, NK cell apoptosis panel **B.**, NK cell depletion panel **C.** and ML-2 cell damage panel **D.** Also, following the first incubation with ML-2 cells, STNK and LTNK cells were magnetically sorted from cell preparations and reincubated overnight with ML-2 cells at 37°C. Then, cells were stained with FITC-annexin-V and PI and analyzed by flow cytometry. ML-2 cells were identified by posting an electronic gate on cells with the highest forward and side scatters panel **E.**

To determine the extent of damage induced by ML-2 cells in STNK and LTNK cells both types of cells were subjected to 2-consecutive rounds incubation, the extent of damage induced in STNK cells by ML-2 cells was significantly lower than that induced following the first incubation with ML-2 cells (Figure 3, panels B, and C). The reduced susceptibility of ML-2 cell-induced NKCAs of STNK cells was associated with a decreased anti-leukemia activity (Figure 3D, upper and lower left panels). In contrast, following both the first and second incubation, with ML-2 cells, LTNK cells were resistant to ML-2 cells, and were effective in inducing damage of ML-2 cells (Figure 3D, upper and lower right panels) suggesting that ML-2 cells reduce the frequency of STNK effector cells following their first incubation. Also, following their first incubation with IL-2 cells, viable STNK and LTNK cells, sorted from the NK and ML-2 cell preparation, induced a similar extent of ML-2 cell-damage (Figure 3, panel E). These results suggest that STNK cells are composed of at least two subpopulations: one susceptible and the other one resistant to ML-2 cell induced NKCAs.

### Inhibition by LTNK cells of ML-2 cell growth in CB17 scid mice

**Figure 4:**
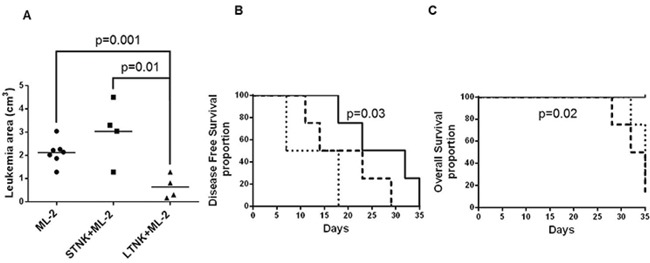
Long-term cultured NK cells inhibit subcutaneous ML-2 cell growth in CB17 scid mice ML-2 cells (4 × 10^6^) were mixed and injected subcutaneously, in the right flank of CB17 scid mice, without (N=8) or with 12 × 10^6^ IL-2 stimulated STNK cells (N=4) or 12 × 10^6^ IL-2 stimulated LTNK cells (N=4). Leukemia growth was monitored up to six weeks. Panel **A.** shows scatter plot analysis of tumor volumes of subcutaneous leukemia following a 37-day post injection. Panels **B.** and **C.** show DFS and OS, as indicated, in which CB17 scid mice were injected with ML-2 cells (dashed lines), ML-2 cells mixed with STNK cells,(dotted lines) and ML-2 cells mixed with LTNK cells (solid lines).

To compare the ability of LTNK and STNK cells to inhibit the subcutaneous growth of leukemia cells, ML-2 cells were mixed with LTNK cells or STNK cells and injected subcutaneously into CB17-scid mice. ML-2 cells injected into mice without NK cells were used as a reference control. LTNK cells significantly inhibited leukemia cell growth (*P* = 0.001) while STNK cells did not (Figure 4A). In addition, LTNK cells significantly prolonged disease free survival (DFS) (*P* = 0.03) and overall survival (OS) (*P* = 0.01) of leukemia bearing mice as compared to STNK cells (Figure 4, 4B, and 4C). These data suggest that LTNK cell treatment is associated with a favorable clinical course of the disease.

### Resistance to AML cell-induced NKCAs in the long-term culture setting is IL-2 independent and is in part mediated by MMPs inhibited by TIMP3

To investigate whether RNA transcription is involved in the generation of NK cell resistance to NKCAs, we tested the effect of actinomycin D on LTNK cell culture. We utilized 10 ng/ml of actinomycin D since this concentration did not affect the expression of CD16 and the percentage of NK cells present in PBMC preparations. Table 1 shows that following a 3-day culture with ML-2 cells, STNK cells underwent NKCAs and following a 10-day culture, LTNK cells became NKCA resistant. In contrast, ML-2 cells induced a significant down-regulation of CD16 expression (*P* = 0.004) and a trend toward significant reduction in the NK cell number (*P* = 0.09) in the PBMC preparations cultured in the presence of actinomycin D. These results suggest that NK cell RNA transcription is involved in the generation of AML cell-induced NKCA.

**Table 1:** Actinomycin D restores leukemia cell-induced NKCA in the long term-cultured NK cells

	% of CD16 down regulation		
	3 days	10 days	10 days+actinomycin D
Exp 1:NK + ML-2	49	0	24
Exp 2:NK + ML-2	36	7	53
Exp 3:NK + ML-2	29	13	31
Exp 4:NK + ML-2	25	9	42
Statistics	34.7±10.5	7.2±5.4	37.5±12.7

	% of NK cell frequency inhibition		
	3 days	10 days	10 days+actinomycin D
Exp 1:NK + ML-2	65	13	69
Exp 2:NK + ML-2	70	33	89
Exp 3:NK + ML-2	59	0	10
Exp 4:NK + ML-2	32	0	31
Statistics	56.5±16.9	11.5±15.5	49.7±35.7

Following a 3 day of stimulation with IL-2, aliquots of PBMCs were harvested and utilized for the assessment of NKCAs in the presence or absence of ML-2 cells. At the same time, actinomycin D (10 ng/ml) was added to the ongoing culture of PBMCs. On day 10, PBMCs were harvested and incubated for 18 hours in the presence or absence of ML-2 cells. NKCAs were evaluated by flow cytometry utilizing fluorescent conjugated anti-CD56, anti-CD16 and anti-CD3 mAbs. For statistical analysis: % of CD16 down-regulation and % of NK cell frequency inhibition were calculated by dividing CD16MFI and % of NK cell values obtained from PBMCs cultured in the presence of ML-2 cells by CD16 MFI and % of NK cells values obtained from PBMCs cultured in the absence of ML-2 cells x100 respectively. For CD16 down-regulation we obtained: 3 days vs 10 days: *P* = 0.0035, 10 days vs 10 days + actinomycin D: *P* = 0.004, 3 days vs 10 days+actinomycin D: *P* = 0.7. For % of NK cell frequency inhibition we had the following results: 3 days vs 10 days: *P* = 0.0079, 10 days vs 10 days + actinomycin D: *P* = 0.09, 3 days vs 10 days + actinomycin D: *P* = 0.7.

**Figure 5:**
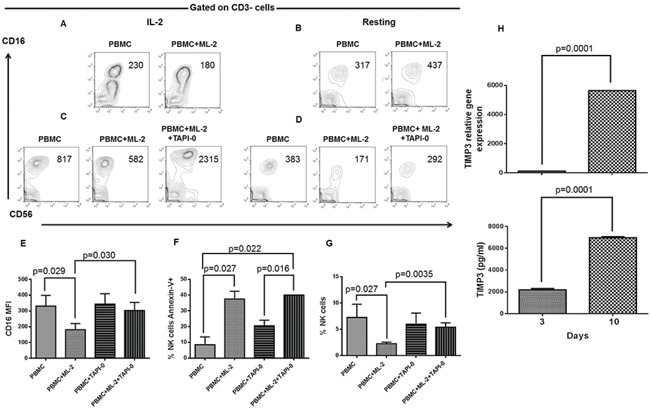
Inhibition of AML cell-induced CD16-down-regulation and NK cell depletion by TAPI 0 defines a role for TIMP3 in the resistance of LTNK cells to AML-cell-induced NKCAs Panels **A.** and **B.** Following a 10-day of culture, IL-2 (200 U/ml), post-activated and resting PBMCs were harvested and tested for NKCAs in the presence of ML-2 cells at a ratio of 1 PBMC to 1 ML-2 cell. Following a 18-hour incubation, NK cells were analyzed for the expression of the indicated cell surface molecules by flow cytometry. Numbers included in the figure quadrants indicate CD16 MFI. Panel **C.** and **D.** Following a 3-day of culture, IL-2 (200U/ml), post-activated and resting PBMCs were harvested and challenged with or without ML-2 cells. Left and right panels show the effects of TAPI-0 (10 mM) on CD16 cell surface expression of NK cells contained in PBMCs after 18 hours of culture in the presence or absence of ML-2 cells. Numbers in quadrants indicate CD16 MFI. This figure shows 1 representative experiment out of 3 performed with similar results. Following a 3-day of culture, IL-2 post-activated and resting PBMCs were harvested and incubated in the presence or absence of TAPI-0 and tested for NKCAs in the presence of ML-2 cells with or without TAPI-0 at a ratio of 1 PBMC to 1 ML-2 cell. Following an 18-hour incubation, NK cells were tested for CD16 expression panel **E.** apoptosis panel **F.** and NK cell depletion **G.** This figure shows a cumulative analysis of results obtained from 3 experiments independently performed. LD-PBLs enriched NK cells(>50% of CD3-CD56+CD16± cells) were grown in CM. At the indicated times, cells and culture supernatants were harvested. NK cells were further purified by magnetic sorting. Total RNA isolation and culture supernatants were utilized for the analysis of TIMP3 gene expression (upper panel **H.** and the assessment of soluble TIMP3 (lower panel H) respectively. Gene expression of TIMP3 was evaluated by q(RT)-PCR utilizing beta-actin gene expression as a reference while soluble TIMP3 was measured by ELISA. This figure shows 1 representative experiment out of 3 performed with similar results.

MMP activation during NK cell/leukemia cell conjugation has been recently shown to cause CD16 down-regulation in resting NK cells. Consistent with this result, chemical inhibitors of MMPs have been shown to inhibit CD16 down-regulation induced by AML cells.[11] This finding provided us with the rationale to test whether TIMP3, a potent endogenous MMP inhibitor is involved in the inhibition of NKCA induction by AML cells. To investigate this possibility, we examined the IL-2 stimulated NK cells. As shown in Figure 5, (panel A and B), following a 10-day culture, IL-2 stimulated and resting LTNK cells were not significantly susceptible to ML-2 cell-induced NKCAs. In addition, TAPI-0 inhibited the down-regulation of CD16 on STNK cells, (Figure 5, panel C) and this effect was also detected when PBMCs cultured without IL-2 were used as targets (Figure 5, panel D). Notably, TAPI-0, at the concentration utilized did not alter the basal expression of CD16 and the percentage of NK cells. It reduced leukemia cell-induced CD16 down-regulation (Figure 5, panel E) and NK cell depletion (Figure 5, panel G) but did not affect leukemia cell-induced NK cell apoptosis (Figure 5, panel F). These results suggest that TIMP-3 inhibits ML-2 cell-induced CD16-down-regulation and NK cell depletion but does not inhibit NK cell apoptosis.

### Role of TIMP3 in LTNK cell resistance to AML cell-induced NKCAs

The ability of TAPI-0, to inhibit the induction of CD16-down-regulation and cell depletion by AML cells raised the possibility that TIMP3 may play a role in the resistance of LTNK cells to AML cell-induced NKCAs. To prove this possibility, we measured the expression level of TIMP3 gene and protein in LTNK cells. LTNK cells displayed levels of TIMP3 gene expression (Figure 5, upper panel H) and protein shedding (Figure 5, lower panel H), in their culture supernatant, significantly higher than those found in STNK cells. Thus, in LTNK cells cultured at 37°C, the level of TIMP3 gene expression in NK cells was about 50 times higher than that in NK cells following a 3-day culture at 37°C. Similarly, following a 3 and a 10-day culture at 37°C, NK cells produced 2192±69 and 6963±59 pg/ml of soluble TIMP3, respectively. These results suggest that TIMP3 is associated with the reduced susceptibility of LTNK cells to AML cell-induced NKCAs.

## DISCUSSION

The results, we have described, indicate that AML cells induce abnormalities in a fraction of both CD16+ and CD16- NK cell-subsets. They include CD16 down-regulation, NK cell apoptosis, and NK cell depletion. NKCAs are reversed by long-term culture of NK cells at 37°C; this is associated with an increased ability of LTNK cells to inhibit subcutaneous ML-2 cell growth, in CB17 scid mice, leading to a DFS and OS prolongation. The resistance to NKCAs by LTNK cell culture which involves RNA transcription is associated with TIMP3 over-expression *in vitro*.

Our results show that STNK cells are not homogenously damaged by AML cells, but they harbour NK cell that are not damaged by AML cells. The identification of these cells is not readily apparent; although, their phenotypic and functional characterization may contribute to improving the therapeutic efficacy of immunotherapy of AML cells with NK cells.

In agreement with the results reported by Jewett et al. and by Grzywacz et al.[8, 11] we have found that leukemia cells cause CD16 down-regulation on STNK cells. This defect is likely to reflect MMP activation induced by NK cell-AML cell conjugation. As a result, CD16 antigen is trimmed and shed from NK cell surface.[11] The key role of MMPs in the generation of this abnormality is also supported by the ability of MMP drug inhibitors to reverse the CD16 down-regulation in NK cells induced by leukemia cells and phorbol ester activation.[11, 17, 18] We have shown for the first time that biological MMP inhibitors such as TIMPs can inhibit CD16 down-regulation. We have focused our attention on TIMP3 because of its unique ability to induce apoptosis in normal and tumor cells *in vitro*.[19, 20]

CD16 antigen down-regulation is always associated with NK cell apoptosis and depletion. This association has been taken as evidence to suggest that CD16 antigen triggering plays a role in AML cell-induced NKCAs. In support of this possibility, a few studies have shown that cross-linking of CD16 antigen by the corresponding mAbs induces NK cell apoptosis [12] and anergy in Fas+ NK cells. These cells, in the presence of endogenous TNF-α, undergo apoptosis that is inhibited, *in vitro*, by anti-TNF-α mAb; only a minor population of NK cells remains cytotoxic and proliferates in the presence of IL-2. These findings support a role for TNF-α in the induction of NK cell apoptosis.[21] To reinforce the role of CD16 in AML-induced NKCAs, we have shown that cross-linking of CD16 on NK cells by the Fc fragment of SY11B5, a mAb with specificity for CD157 antigen expressed on leukemia cells but not on NK cells, induces CD16 down-regulation and enhances NK cell apoptosis. However, neither FAS nor TNF-α death pathways seem to be involved in the induction of NK cell apoptosis and depletion since NKCAs are not affected by caspase blockade.[14] In addition, the CD56+CD16- NK cell subset undergoes AML cell-induced apoptosis to the same extent as the CD56+CD16+ NK cell subset. Furthermore, TAPI-0 reduced CD16 down-regulation and NK cell depletion but did not inhibit NK cell apoptosis suggesting that TIMP3 may not play a role in AML cell-induced NK cell apoptosis.

Because of the potential role of CD16 antigen in the generation of AML cell- induced NKCAs, we investigated whether the length of the *in vitro* incubation of NK cells with IL-2 causes changes in CD16 cell surface expression, STNK cells express a significantly higher CD16 expression level than LTNK cells. The mechanism underlying this difference is not readily apparent. One might speculate that changes over time of CD16 expression on NK cells are independent of MPP inhibition by TIMP3.[22, 23] Two lines of evidence support this hypothesis. First, TAPI-0 did not up-regulate CD16 expression on IL-2 stimulated STNK cells; second TAPI-0 decreased TIMP3 gene expression and protein secretion in IL-2 stimulated LTNK cells as compared to resting NK cells (data not shown). It is tempting to speculate that a population of NK cells with low or intermediate CD16 expression may be selected from a long-term NK cell culture. These results prompted us to test, *in vitro* and *in vivo*, the effect of AML cells on STNK and LTNK cells. LTNK cells become resistant to leukemia cell-induced NKCAs and this phenomenon is associated with an apparent anti-AML cell activity, *in vivo*, leading to AML cell growth inhibition in immunodeficient mice and, *in vitro*, TIMP3 over-expression in NK cells. It is important to highlight that subcutaneous injection of IL-2 stimulated STNK cells in the presence of ML-2 cells, in CB17 scid mice, does not inhibit leukemia growth and has no effect on the clinical course of the disease. In this context, we can hypothesize that NKCA induced by ML-2 cells on STNK cells decreases their anti-leukemia potential. The lack of anti-leukemia activity of STNK cells, *in vitro and in vivo*, may reflect a reduction in the frequency of the functionally active NK cells due to the harmful effects of AML cells on STNK cells but not on LTNK cells. NKCA resistance to cancer cells became detectable following a 7-day culture and reached its peak on day 10 of NK cell culture. Based on this information one may speculate that the reduced expression of cell surface CD16 antigen may protect LTNK cells from AML cell-induced NKCAs. This kinetics is compatible with the possibility that changes in RNA transcription and protein synthesis are involved in the development of LTNK cell resistance to ML-2 cells. Indeed, LTNK cells cultured in the presence of actinomycin D for 10 days lost their resistance to ML-2 cells indicating that ML-2 cell-induced NKCAs involve RNA transcription.

TAPI-0 has inhibitory activity on a few MMPs including ADAM9, ADAM17, and MMP-1, 3, 9, and 13. [22, 23] Since TAPI-0 inhibited CD16 down-regulation and NK cell depletion, MMP inhibitors may be utilized to reduce STNK cell susceptibility to AML cell-induced NKCAs. On the other hand, Romee et al. demonstrated that a disintegrin and ADAM17 regulate CD16 expression on NK cells.[24] Furthermore, Wiernik et al. showed that ADAM17 inhibition enhances the cytotoxic activity of NK cells against myeloid leukemia cells by the anti-CD16xanti-CD33 bispecific antibody. [25] The high resemblance of TIMP3 and TAPI-0 in their functional activity suggests that TIMP3 could play a role in the induction of the mechanism by which LTNK cells become resistant to AML cell-induced NKCAs. LTNK cells may acquire the ability to increase TIMP3 gene transcription and protein release in the culture supernatant. Then, soluble TIMP3 could interfere with MMP activation mediated by malignant cells.

Our data suggest that mechanism(s) underlying resistance of NK cells could be utilized to improve haploidentical or allogeneic LTNK and STNK-cell-based immunotherapy for hematological malignancies. In this context, the optimization of methodologies aimed at producing a good manufacturing practice expansion of LTNK cells may lead to their clinical implementation in the therapy of AML.[1–3] Also, the inhibition of AML cell-induced STNK cell abnormalities by drug mimicking the effects of TIMP3 such as TAPI-0[26] on STNK cells may allow STNK cells to be utilized to fight AML disease.

## MATERIALS AND METHODS

### Antibodies and reagents

Fluorescein isothiocyanate (FITC)-conjugated mouse anti-human-CD16, phycoerythrin-(PE)-conjugated anti-CD16, phycoerythrin-cyanine-dye5 (PE-Cy5)-conjugated anti-CD3, allophycocyanin (APC)-conjugated anti-CD56, isotype-matched control monoclonal antibodies (mAbs) and FITC-annexin-V and propidium iodide (PI) were purchased from BD Bioscience (San Jose, CA). Anti-human CD157 (SY11B5) mAb was purchased from eBioscience (San Diego, CA). RPMI 1640 medium was purchased from Lonza (Milan, Italy). Recombinant IL-2 was obtained from Chiron Corporation (Emeryville, CA).

### Cell lines

The human myeloid leukemia cell lines ML-2, THP-1 and U937 were cultured in RPMI 1640 medium supplemented with 10% fetal bovine serum (FBS), glutamine (10 mg/ml), penicillin (10U/ml) and streptomycin (10 mg/ml). Cells are part of our cell line laboratory collection. The identity of the ML-2 and U937 cell lines is monitored by a phenotypic analysis of cell surface markers. The latter is identified as CD45+HLA-A2-chondroytin sulphate proteoglycan (CSPG) 4+ while the former is identified as CD45+HLA-A2+CSPG4+. The identity of the THP-1 cells is monitored by the analysis of cell surface expression of CD45 and Fc receptor and by HLA class II allele genotyping including DRW1 and DRW2.

### Isolation of peripheral blood mononuclear cells (PBMCs)

PBMCs isolated by Ficoll-Hypaque density gradient centrifugation from buffy coats obtained from the University of Tor Vergata Blood Bank donors. Low-density peripheral blood lymphocytes (LD-PBLs) were isolated as previously described.[13] PBMCs and LD-PBLs were activated with IL-2 (200 U/mL) for 3-16 days at 37°C in RPMI 1640 medium supplemented with 10% FBS; this medium is referred to as a complete medium (CM). CD56^+^ cells and NK cells were isolated utilizing anti-CD56 magnetic beads and the NK cell isolation kit, respectively (Miltenyi Biotec, Auburn, CA).

### Flow cytometry analysis

PBMCs or magnetically sorted CD56^+^ cells were mixed with leukemia cells at different E/T ratio. Following a 5-18-hour incubation at 37°C in a 5% CO_2_ atmosphere, cells were harvested, incubated for 30 min at 4°C with PE-conjugated anti-CD16 mAb, PE-Cy5-conjugated anti-CD3 mAb, APC-conjugated anti-CD56 mAb, Then, cells were incubated for 15 min at room temperature, in the dark, with FITC-annexin-V and analyzed as already described.[14] Results were analyzed utilizing the BD Cell Quests and the Tree Star Inc. flowJo software.

### Cytotoxicity assay

The standard ^51^Cr-release cytotoxicity assay was performed as described.[15] Maximum Cr release was measured by incubating target cells with 5% triton-X-100. Percent of specific lysis was calculated utilizing the formula: (experimental release-spontaneous release) / (maximum release-spontaneous release) × 100. The spontaneous release was less than 10%.

### Mice

Ten-week-old male CB17 SCID mice were purchased from Charles River Laboratories (Lecco, Italy) and housed in laminar flow cages. Mice were fed with sterile food and water. Mouse age at the beginning of the experiments ranged between 20-22 weeks. Mice endogenous NK cell activity was inhibited by an intraperitoneal injection of 50 ml of anti-asialo GM1 antiserum (Wako, Chemicals, Richmond, VA,) on day -3, 0, +14 and +21 utilizing as a reference time that of a subcutaneous injection of ML-2 cells or ML-2 plus NK cells.

### *In vivo* anti-leukemia activity of short term and long term NK cells

PBMCs were cultured in CM at 37°C. Following a 3-day and a 10-day culture, PBMCs were harvested, and NK cells were negatively sorted (95% purity). Then ML-2 cells (4 × 10^6^) were mixed with 3-day cultured NK cells (12 × 10^6^), or with 10-day cultured NK cells (12 × 10^6^), hereafter referred to as short term NK (STNK) cells, and long-term NK (LTNK) cells, respectively. Cell mixtures were resuspended in PBS supplemented with matrigel (Corning, Tewksbury, MA). Following a 30-min incubation at 37°C, 4 mice were injected with an ML-2-STNK cell suspension and 4 with an ML-2-LTNK cell suspension. Eight mice were injected with an ML-2 cell suspension and used as a control. Subcutaneous ML-2 cell growth was monitored 3 times weekly with calipers. Tumor volume (TV) was calculated using the formula: TV (cm3)=4/3p r^3^, where r=(length + width)/4. When tumor volume exceeded 2 cm^3^, a mouse was sacrificed.

### Gene expression analysis

Following TRIzol based isolation (Life Technologies Italia, Monza, Italy) from cells, total RNA was subjected to reverse transcription using M-MLV reverse transcriptase (Invitrogen, Carlsbad, CA). Quantitative RT-PCR of TIMP3 and β-actin was performed using the TaqMan universal PCR master mix. Primers and probes of TIMP3 were purchased, from Life Technologies (Carlsbad, CA) as a kit assay product, Hs00165949_m1 (TIMP3). Gene expression was quantified as previously described.[16] Gene expression was normalized on the b-actin housekeeping gene.

### Statistical analysis

Results were analyzed using a Mann-Whitney test or a paired-*T*-test. DFS and OS results were evaluated by a Kaplan-Meyer analysis. Differences were considered significant when the *p* value was < 0.05.
